# A Nuclear Family A DNA Polymerase from *Entamoeba histolytica* Bypasses Thymine Glycol

**DOI:** 10.1371/journal.pntd.0000786

**Published:** 2010-08-10

**Authors:** Guillermo Pastor-Palacios, Elisa Azuara-Liceaga, Luis G. Brieba

**Affiliations:** 1 Laboratorio Nacional de Genómica para la Biodiversidad, CINVESTAV, Irapuato, México; 2 Posgrado en Ciencias Genómicas, Universidad Autónoma de la Ciudad de México, México Distrito Federal, México; Bose Institute, India

## Abstract

**Background:**

Eukaryotic family A DNA polymerases are involved in mitochondrial DNA replication or translesion DNA synthesis. Here, we present evidence that the sole family A DNA polymerase from the parasite protozoan *E. histolytic*a (EhDNApolA) localizes to the nucleus and that its biochemical properties indicate that this DNA polymerase may be involved in translesion DNA synthesis.

**Methodology and Results:**

EhDNApolA is the sole family A DNA polymerase in *E. histolytica*. An *in silico* analysis places family A DNA polymerases from the genus *Entamoeba* in a separate branch of a family A DNA polymerases phylogenetic tree. Biochemical studies of a purified recombinant EhDNApolA demonstrated that this polymerase is active in primer elongation, is poorly processive, displays moderate strand displacement, and does not contain 3′–5′ exonuclease or editing activity. Importantly, EhDNApolA bypasses thymine glycol lesions with high fidelity, and confocal microscopy demonstrates that this polymerase is translocated into the nucleus. These data suggest a putative role of EhDNApolA in translesion DNA synthesis in *E. histolytica*.

**Conclusion:**

This is the first report of the biochemical characterization of a DNA polymerase from *E. histolytica*. EhDNApolA is a family A DNA polymerase that is grouped into a new subfamily of DNA polymerases with translesion DNA synthesis capabilities similar to DNA polymerases from subfamily ν.

## Author Summary

Genotoxic agents like ultraviolet radiation, alkylating compounds and reactive oxidative species have the potential to originate DNA lesions that are not bypassed by replicative DNA polymerases. Eukaryotic organisms contain a specialized subset of DNA polymerases capable of translesion DNA synthesis. These DNA polymerases belong to DNA polymerases from families A, B, and Y. In this work, we characterized the sole family A DNA polymerase of the parasitic protozoa *E. histolytica,* EhDNApolA. The biochemical characterization of recombinant EhDNApolA indicates that this protein is an active DNA polymerase able to primer extension and moderate strand displacement. The ability of EhDNApolA to faithfully incorporate dATP opposite thymine glycol, and its nuclear localization indicates that this polymerase may have a role in translesion DNA synthesis. *E. histolytica* is exposed to oxidative stress during tissue invasion by phagocytes. Understanding DNA metabolism in *E. histolytica* is important because this parasite has shaped some metabolic pathways by horizontal gene transfer, infects approximately 50 million people annually, and is the second leading cause of death among protozoan diseases.

## Introduction

DNA replication and translesion DNA synthesis in eukaryotes is accomplished by a battery of DNA polymerases. For instance, the genome of *Homo sapiens* contains 15 DNA polymerases divided into four families: A, B, X, and Y according to their amino acid sequence homology [1]–[3]. Nuclear replicative DNA polymerases δ and ε · belong to family B, whereas DNA polymerases involved in translesion DNA synthesis are present in all four families.

*Entamoeba histolytica* is a parasitic protozoa which causes amebic dysentery and liver abscess [4]. In comparison to eukaryotes that contain DNA in organelles like mitochondria or chloroplasts. *E. histolytica* is an early branching eukaryote in which its mitochondria diverged to form an organelle with no detectable DNA. This organelle is dubbed mitosome [5], [6], and although its function is not definitively established, experimental evidence suggests a role in sulfate activation [7] and oxygen detoxification [8]. Thus, the 24 Mbp genome o*f E. histolytica* is exclusively nuclear and it encodes several putative DNA polymerases (Table S1) [9]. As an eukaryotic organism, the genome of *E. histolytica* is expected to be replicated by DNA polymerases δ and ε. Although a gene encoding DNA polymerase ε is not present in the current genome annotation of *E. histolytica*, a gene encoding DNA polymerase δ is present. *E. histolytica* contains homologs of Rev 1 and Rev 3 proteins, that compose the principal DNA polymerase involved in translesion synthesis of thymine dimers: DNA pol ζ [10], [11]. In addition, the genome of *E. histolytica* contains five DNA polymerases which share high sequence homology with DNA polymerases from autonomous replicating elements found in other protozoa and with the well-characterized DNA polymerase from bacteriophage φ29[12]. *E. histolytica* also contain one family A DNA polymerases in its genome. Family A DNA polymerases are modular enzymes consisting of three independent domains: a N-terminal 5′-3′ exonuclease domain, a 3′-5′ exonuclease domain, and a C-terminal polymerase domain [1], [13], [14]. Crystal structures of family A DNA polymerases revealed a modular organization of the polymerase domain and its division into three subdomains: palm, fingers, and thumb, which together form a cleft that binds the primer-template [15]. Family A DNA polymerases contain three conserved motifs: A, B, and C in the polymerization domain [16]. Motifs A and C are located at the palm subdomain and contain two carboxylates involved in the coordination of two metal ions involved in the nucleophilic attack of the incoming deoxynucleotide to the 3′ OH of the primer strand [13]. Motif B is located at the fingers subdomain and is involved in positioning the template strand into the polymerase active site [15]. In eukaryotes, family A polymerases are involved in the replication of mitochondrial and chloroplast genomes [17], [18]. The archetypical DNA polymerase in eukaryotes is DNA polymerase γ, which is the replicative mitochondrial DNA polymerase. Besides DNA polymerase γ, vertebrates contain two other family A DNA polymerases: DNA polymerase ν and DNA polymerase θ. In contrast to DNA polymerase γ, the localization of these polymerases is nuclear. Human DNA polymerases ν and θ are capable of translesion DNA synthesis and they have a role in DNA repair [19]–[24].

In this work, we report the initial characterization of the sole family A DNA polymerase from *E. histolytica* (EhDNApolA). We propose a role of this DNA polymerase in translesion DNA synthesis of oxidative lesions like 8-oxo guanosine and thymine glycol. These lesions may be generated by the oxidative environment of the colonic tissue and the constant insult of the reactive oxygen species produced by phagocytes during *E. histolytica* pathogenesis.

## Materials and Methods

### Phylogenetic analysis and structural modeling of EhDNApolA

To identify putative family A DNA polymerases in *E. histolytica*, we initially used the amino acid sequence of the Klenow fragment of *E. coli* (Protein Data Bank accession code: 1KFS) to blast the Pathema database (http://pathema.jcvi.org/Pathema/).

The phylogenetic tree was constructed using the amino acid sequences of family A DNA polymerases of representative mitochondrial DNA polymerases, bacteriophage DNA polymerases, DNA polymerases ν, and bacterial DNA polymerases. The amino acid sequences of these proteins were aligned using the program ClustalW [25]. The catalytic amino acids of motifs A, B, and C, were conserved through the alignment. This sequence alignment was used to construct a dendogram with the Neighbor-Joining method of the Molecular Evolutionary Genetic Analysis (MEGA) software [26]. The robustness of the dendogram was assessed by bootstrap analysis of 1000 replicates.

To build the structural model of EhDNApolA, the amino acid sequence of EhDNApolA was structurally aligned with the amino acid sequence present in the crystal structure of Klenow fragment (Protein Data Bank accession code: 1KFS) [27], using the program Molecular Operating Environment (MOE). As Klenow Fragment contains 605 amino acids and EhDNApolA has 657, the gaps between the two aligned proteins were built according to the peptide library present in the MOE database. Twenty models were generated and each model was minimized using the CHARMM27 force field.

### *E. histolytica* cultures

Trophozoites of HM1:IMSS strain were axenically cultured in TYI-S-33 medium supplemented with 15% of bovine serum [28] at 37°C and used in logarithmic growth phase for all experiments.

### Cloning of EhDNApolA gene

The open reading frame of EhDNApolA was amplified by PCR from genomic DNA of *E. histolytica* strain HM1:IMSS. To allow directional cloning, the sense oligonucleotide (5′-ggttgg ggatcc atg gaa aaa aca cca aga aat tct-3′) contained a *BamH* I restriction site (underlined) and the antisense oligonucleotide (5′-ggttgg aagctt tta att caa gtt gta agg atg aag-3′) contained a *Hind* III restriction site (underlined). PCR was carried out using 150 ng of genomic DNA, 25 pmol of each oligonucleotide, and 125 µM of each dNTP. The amplified product was simultaneously digested with *BamH* I and *Hind* III and ligated into the pCOLD I vector (Takara). The ligation mixtures were transformed into an *E. coli* DH5α strain. Plasmidic DNA was analyzed using restriction mapping and confirmed by DNA sequencing. Cloning of the open reading frame of EhDNApolA in the pCOLD I vector confers a 6-His tag at the N terminus of the recombinant protein.

### Production of anti-EhDNApolA antibodies

Seven Balb/c mice were bled and tested for their response to total protein extracts of *E. histolytica*. Five mice did not present any response and were inoculated with a peptide corresponding to residues 286 to 297 of the thumb subdomain of EhDNApolA (HKIEMETKKIIG). The mice were immunized with 150 µg of the peptide combined with Freund's adjuvant. Six weekly bursts were applied and the reactivity of each mouse was assessed using recombinant EhDNApolA. After six weeks of immunization, the immune sera was collected, purified, and stored at −20°C.

All animal work was conducted according to the legislation enforced in México (NOM-062-ZOO-1999) and by CINVESTAV's committee for animal care and use. The Mexican legislation is based on the Guide for the Care and Use of Laboratory Animals, NRC.

We tested the antibodies for their response and specificity in total extracts of *E. histolytica* strain HM1:IMSS and against recombinantly induced EhDNApolA. For Western blot assays, we used total, nuclear, and cytoplasmic extracts from *E. histolytica* strain HM1:IMSS prepared as previously described [29]. Protein extracts were separated using a 15% SDS-PAGE gel and transferred onto a nitrocellulose membrane. The membranes were incubated with a 1 to 2000 dilution of the purified immune sera and an anti-actin antibody [30] in 1% nonfat dry milk, 0.05% Tween-20 in PBS 7.4 for 2 hours. The reactivity was detected using peroxidase conjugated secondary antibodies (1 to 2000 dilution) with the ECL Plus detection kit (GE Healthcare). As a control, we used antibodies against actin and CBP-B previously characterized.

### RT-PCR assays

cDNA was synthesized using 1 µg of total *E. histolytica* RNA with an oligo(dT) adaptor. The RT-PCR reactions contained 0.5 µl of cDNA and 15 pmol of each specific oligonucleotide combination. The segment corresponding to motif A was amplified using the sense oligonucleotide 5′-agagacttattattacacat3-' and antisense oligonucleotide, 5′-attctttttaagccaatgtgc-3′. Motif C was amplified using the sense oligonucleotide; 5′-ttacattcaagttgggtaggt-3′ and antisense oligonucleotide 5′-aacagtaactacaacaggaac-3′. The actin control was amplified with the sense oligonucleotide 5′-aag ctg cat caa gca gtg aa-3′ and antisense 5′-gga atg atg gtt gga aga gg -3′. RT-PCR products were separated by gel electrophoresis in 1.5% agarose gels, stained with ethidium bromide, and visualized with a standard UV transilluminator.

Semi-quantitative RT-PCR assays were performed using total cellular RNA isolated from *Entamoeba histolytica* grown in basal culture conditions using SV *Total* RNA Isolation System (Promega Madison, WI, USA). The amount of total or messenger RNA isolated from the cells was quantified using an ND-1000 spectrophotometer (*NanoDrop*, Fisher Thermo, Wilmington, DE, USA). cDNA was synthesized using gene-specific primers. 1 µg of total RNA was added to a reaction containing 625 mM EhDNApolA motif A antisense oligonucleotide or actin antisense oligonucleotide, 0.5 mM of the deoxynucleotide triphosphates, 1 unit of RNasin Ribonuclease Inhibitor, 1 ml of ImProm-II™ Reverse Transcriptase (Promega Madison, WI, USA) and RNase-free water to 20 µl. Reactions were incubated at 25°C for 5 min, then at 42° for 60 min followed by 75°C for 15 min, to inactivate the reverse transcriptase. PCR was performed using EhDNApolA or actin specific sprimers to amplify cDNA segments of 168 or 192 bp in length respectively, with the estimated primer melting temperature of 61.5 or 52°C. RT-PCR products were separated by gel electrophoresis in 1% agarose gels, stained with ethidium bromide, and visualized with a standard UV transilluminator.

### Protein expression and purification

The pCOLDI-EhDNApolA construct was transformed into an *E. coli* BL21 DE3-Rosseta II strain. Transformants were inoculated in 100 ml of LB supplemented with 100 µg/ml of ampicilin and 35 µg/ml of chloramphenicol and used to inoculate 2 liters of LB. This culture was grown at 37°C until it reached an OD_600_ of 0.6. The culture was incubated in ice for 30 minutes and IPTG was added to a final concentration of 0.5 mM. Induction proceeded for 16 hours at 16°C. The cell pellet was harvested by centrifugation at 6,500 rpm. Cell lysis was carried out using a French press in a buffer containing 50 mM potassium phosphate pH 8, 300 mM NaCl, and 1 mM PMSF. The lysate was centrifuged at 17,000 rpm for 30 minutes at 4°C. The soluble fraction was filtrated and the recombinant EhDNApolA was purified using a Ni^2+^-NTA affinity chromatography in a previously equilibrated Hi-Trap Column (GE Healthcare). The initial wash consisted of 50 ml of lysis buffer supplemented with 35 mM imidazol and the second wash consisted of 100 ml of lysis buffer supplemented with 50 mM imidazol. Protein elution was carried out in lysis buffer supplemented with 500 mM imidazol. The eluate was dialyzed in a buffer containing 50 mM potasium phosphate pH 7.0, 5 mM β*-*mercaptoethanol (BME), 50 mM NaCl, 2 mM EDTA and 5% glycerol. To further purify EhDNApolA, the dialyzed protein was loaded into a phosphocellulose column and eluted with a NaCl gradient (100 to 1500 mM). EhDNApolA eluted between 600 to 650 mM of NaCl. The collected fractions were dialyzed in 50 mM potasium phosphate pH 7.0, 1 mM β*-*mercaptoethanol, 150 mM NaCl and 1 mM EDTA and stored at 4°C. Protein samples were run on a 10% SDS-PAGE and stained with Coomassie Brilliant Blue R-250.

### Polymerization substrates

The following oligonucleotides were used to generate double stranded polymerization substrates: a) 45mer template (5′-cct tgg cac tag cgc agg gcc agt tag gtg ggc agg tgg gct gcg-3′) b) 24mer primer (5′-cgc agc cca cct gcc cac cta act-3′); c) 18mer primer (5′-cgc agc cca cct gcc cac-3′); and d) 21mer non-template (5′-ggc cct gcg ctagtgccaagg-3′).100 nmols of the 24mer primer or the 18mer primer were 5′end labeled using T4 Kinase with γ-[^32^]ATP. The probes were purified using the nucleotide removal kit (Qiagen) according to the manufacturer instructions. The polymerization substrates were annealed to a final concentration of 10 nM in 20 mM Tris pH 7.5, 150 mM NaCl.

### DNA binding

A radiolabeled DNA substrate consisting of the 45mer template annealed to the 24mer primer was incubated with increasing concentrations of EhDNApolA (from 0 to 180 nM) in a buffer containing 50 mM NaCl, 10 mM Tris-HCl pH 7.5, 2.5 mM MgCl_2_, 1 mM dithiothreitol (DTT), 1 µg/ml BSA, and 5% glycerol. DNA-protein complexes were resolved through a 6% non-denaturing polyacrylamide gel (PAGE) and electrophoresed at 80 V for 2 h at room temperature in 0.5x TBE buffer. Gels were vacuum-dried and radioactive complexes were detected in a Phosphor Imager apparatus and analyzed using the ImageQuant software (BioRad).

### Polymerization reactions

20 µl polymerization reactions consisted of 20 mM Tris-HCl pH 7.5, 2.5 mM MgCl_2_, 1 mM DTT, 1 µg/ml BSA, 200 fmol primer-template, 60 fmol EhDNApolA. Reactions were stopped with a buffer containing 95% formamide, 1mM EDTA, 0.01% xylene cyanol. Samples were resolved on a 16% polyacrylamide, 8M urea denaturing gels. Quantification of the polymerization products was carried out in a Phosphorimager using ImageQuant software.

### Translesion DNA synthesis

Templates containing 8-oxo guanosine and abasic site were purchased from Oligos Etc. Templates containing 5 S-6R thymine glycol, 5R-6S thymine glycol, cis-syn cyclobutane pyrimidine dimer, and 6-4 photo product were synthesized by Professor Shigenori Iwai's group as previously described [31]. A specific 5′ γ-[^32^]ATP labeled primer was annealed to each template, so the first template base corresponds to each specific lesion. 60, 120 and 240 fmol of EhDNApolA were incubated with 100 fmol of each primer-template at 37°C for 2.5 minutes with 100 µM of each dNTP. Reactions were stopped by adding an equal volume of gel stop/loading buffer. The reactions were run on a 16% denaturing 8 M urea polyacrylamide gel.

### Kinetic analysis

For steady-state kinetic analysis, DNA polymerase activity assays were performed using 2 pmol of duplex DNA incubated with 10 fmol of EhDNApolA and varying dNTP concentrations. The reactions were incubated for 10 minutes at 37°C. Four different DNA duplexes were used to determine the kinetic parameters of each nucleotide opposite to its cognate base. To assure linearity, less than 20% of the substrate was converted to product.

### Confocal microscopy

Trophozoites of *E. histolytica* grown in basal cell culture condition were transferred to glass coverslips. Cells were fixed with 4% paraformaldehyde for 1 hour at 37°C, washed with PBS pH 6.8, permeabilized with 0.5% (v/v) Triton X-100 at 37°C for 60 min, and blocked with 50 mM glycine for 1 h at 37°C and with 1% fetal bovine serum for 15 min. Finally, they were incubated with anti-EhDNApolA antibodies (1 to 75) overnight at 4°C. The cells were washed and conjugated with fluorescein labelled secondary antibodies (Jackson Immuno Research) at 1∶500 dilution. The nucleic acids were stained with DAPI (4′,6′-diamidino-2-phenylindole) washed, and mounted with Vectashield solution (Vector Lab. Burlingame, CA). Light optical sections were obtained through a Nikon inverted microscope attached to a laser confocal scanning system (Leica Microsystems) and analyzed by Confocal Assistant software Image.

## Results

### Identification of a family A DNA polymerase in *E. histolytica*

**Figure 1:**
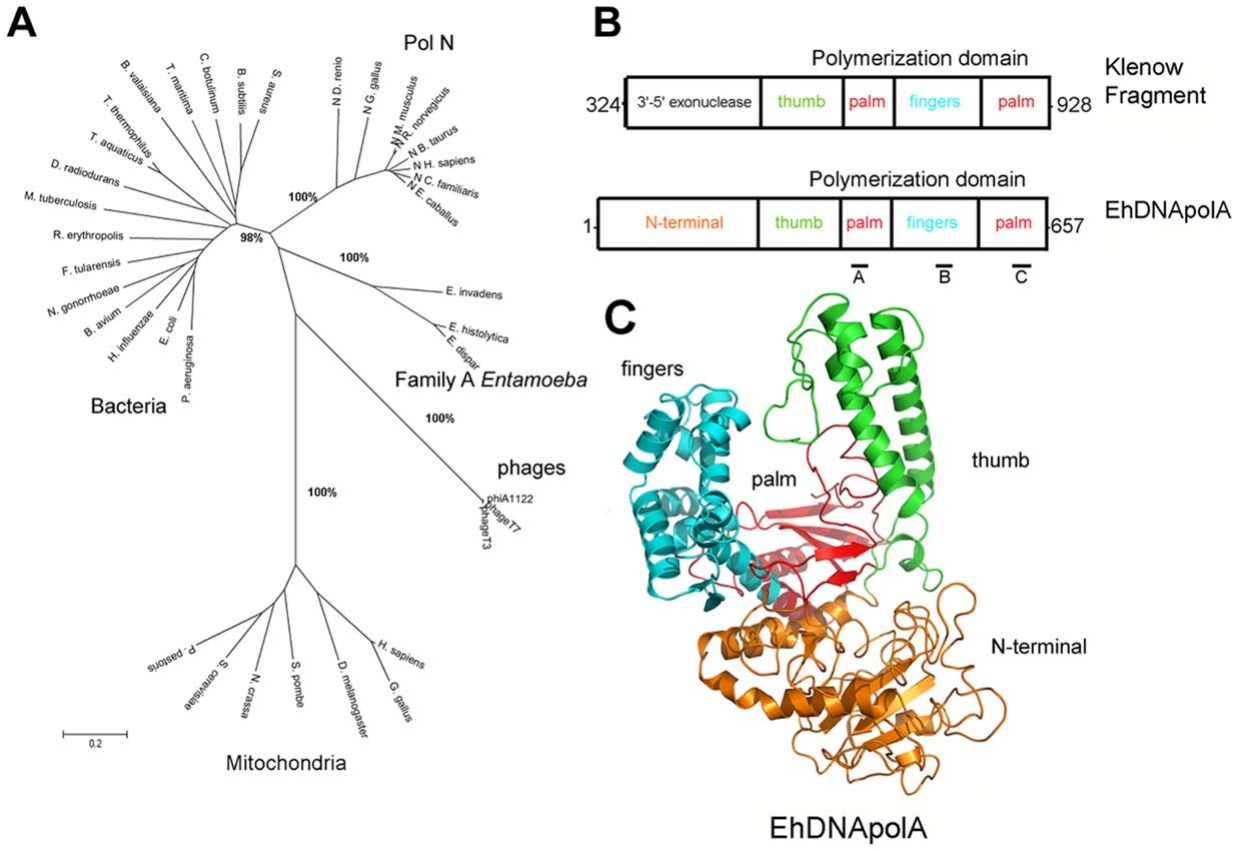
Phylogenetic analysis and structural modeling of *E. histolytica* family A DNA Polymerase. (**A**) Phylogenetic relationship of family A polymerases from different organisms. ClustalW multiple sequence alignment of diverse DNA polymerases were used to construct a phylogenetic tree using the neighbor joining algorithm present in the MEGA program. GenBank accession numbers of each protein are indicated in Table S2. The significance of each branch of the phylogenetic tree is indicated by a bootstrap percentage (**B**) Domain organization of EhDNApolA. EhDNApolA lacks the 3′-5′ exonuclease domain present in Klenow fragment. The polymerization domain of EhDNApolA is organized into three subdomains: thumb, palm, and fingers (**C**) Structural model of EhDNApolA. The N-terminal domain is shown in orange. The polymerization subdomains: thumb, palm, and fingers are colored in green, red, and cyan respectively. The model was constructed using the crystal structure of Klenow Fragment as a template (PDB ID: 1KFS).

A survey of *E.histolytica* genome with the amino acid sequences of Klenow Fragment and representative family A DNA polymerases revealed that this parasite contains a single open reading frame that codes for a putative family A DNA polymerase. This open reading frame is located at locus EHI_073640 and codes for a protein of 657 amino acids with GenBank accession number XP_653960 and 25% amino acid identity to Klenow fragment. In this work we dubbed this putative polymerase EhDNApolA. The predicted amino acid sequence of EhDNApolA was used as query to search for homologous proteins in the genomes of *E. invadens* and *E. dispar*. We found that locus EIN_094210 of *E. invadens* and locus EDI_083910 *of E. dispar* also code for putative family A DNA polymerases with 50% and 88% amino acid sequence identity to EhDNApolA respectively. The lack of a conserved 3′-5′ exonuclease active site in the DNA polymerases of the genus *Entamoeba* indicates that these polymerases are not related to mitochondrial DNA polymerases. A phylogenetic analysis of 37 DNA polymerases (Table S2) positions the DNA polymerases from the genus *Entamoeba* in a separate branch with respect to other subfamily A DNA polymerases. In this division, family A DNA polymerases are grouped into five separate branches or subfamilies (Figure 1A). The high bootstrap value of each branch validates this division. (Figure 1A). Family A DNA polymerase from *Entamoeba* have a clear conservation of the catalytic motifs present in the polymerization domain. (Figure 1B). The disappearance of the exonuclease domain is a common feature in some family A DNA polymerases, including DNA polymerase ν, DNA polymerase θ, and several bacterial polymerases. The crystal structure of Klenow fragment bound to duplex DNA in its exonuclease domain was used as template to build a homology model of EhDNApolA [32]. The structural model of EhDNApolA depicts the modular organization present in family A polymerases. In this model, EhDNApolA adopts a structure that resembles a cupped right hand in which the three subdomains of the polymerization domain (fingers, palm, and thumb) form a DNA binding cleft (Figure 1C). Although this structural model depicts high degree of conservation in the polymerization domain, it should not be interpreted as an experimental structure.

### Over expression and purification of recombinant EhDNApolA

**Figure 2:**
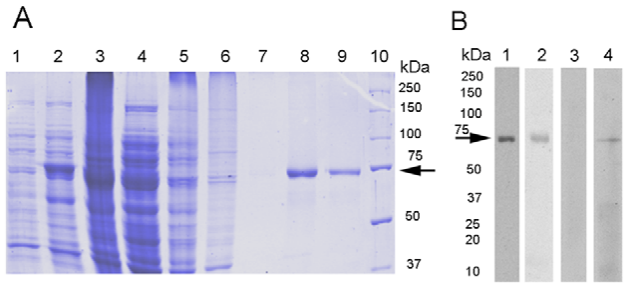
Heterologous expression and purification of *E. histolytica* family A DNA polymerase. (**A**) Coomassie blue stained SDS-PAGE (10%) gel showing the expression and purification of EhDNApolA. Lane1, uninduced pCOLD-EhDNApolA construct; lane 2, IPTG induced sample; lane 3, insoluble fraction; lane 4, soluble lysate; lane 5, nickel agarose flow-through; lane 6, 35 mM imidazol wash; lane 7, 50mM imidazol wash; lane 8, nickel agarose column eluate; lane 9, phosphocelulose column eluate; lane 10, molecular weight standards. (**B**) Detection of recombinant and endogenous EhDNApolA.Recombinant EhDNApolA and total extracts of *E. histolytica* were resolved by SDS-PAGE (15%), electroblotted onto nitrocellulose membrane, and immunoblotted with diverse antibodies. Lane 1, recombinant EhDNApolA treated with commercial anti-6 histidines antibody; lane 2, recombinant EhDNApolA treated with mouse antibodies raised against an epitope of EhDNApolA; lane 3, total protein extracts of *E. histolytica* treated with preimmune serum; lane 4, total protein extracts of *E. histolytica* treated with specific mouse antibodies raised an epitope of EhDNApolA.

In order to test the biochemical properties of EhDNApolA, its open reading frame was cloned into the pCOLD I vector (Takara). Heterologous protein expression was enhanced with the use of the *E. coli* strain BL21- Rosseta II (Figure 2A, lanes 1 and 2, and data not shown). The recombinant EhDNApolA was soluble (Figure 2A, lane 4) and purified nearly to homogeneity using Ni^2+^-NTA affinity chromatography as a first chromatographic step (Figure 2A, lane 8). To assure the purity of the recombinant protein and avoid a possible contamination with endogenous DNA polymerases, we performed a second chromatographic step using a phosphocellulose chromatography. After this step, the recombinant protein was more than 95% pure (Figure 2A, lane 9). Our structural model of EhDNApolA was used to design epitopes to raise polyclonal antibodies. The best epitope candidate was a peptide located at the thumb subdomain (residues 286 to 297) of EhDNApolA. The pre-immune serum did not unveil any reactivity against total extracts of *E. histolytica* (Figure 2B, lane 3) and recombinant expressed polymerase (data not shown). The raised antibodies recognized a single band of 75 kDa in bacterial extracts expressing recombinant EhDNApolA and in total extracts from *E. histolytica* (Figure 2B, lanes 2 and 4). As observed in Figure 2B, the raised polyclonal antibodies were highly specific for EhDNApolA and did not present any cross reactivity that could compromise the localization of EhDNApolA *in vivo*.

### EhDNApol A is a functional DNA polymerase with moderate strand displacement and no exonuclease activity

**Figure 3:**
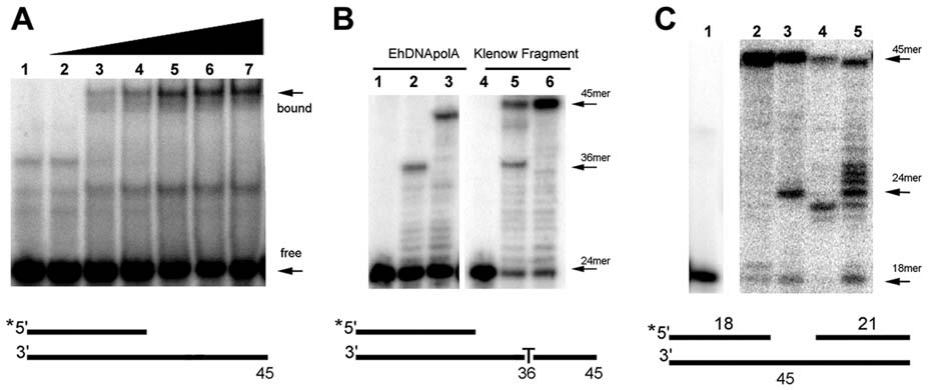
Biochemical characterization of EhDNApolA. (**A**) EhDNApolA binds to double stranded DNA. Increasing concentrations of recombinant EhDNApolA (from 0 to 180 nM) were incubated with a fixed amount of double stranded primer-template. The binding of the EhDNApolA to the primer-template is observed by the formation of a slower migrating complex on a native 6% polyacryalmide gel. (**B**) EhDNApolA is an active DNA polymerase. DNA polymerase activity was measured by the extension of 24mer primer annealed to a 45mer template. Lanes 1 and 4 contained reactions with no added polymerase, EhDNApolA (lanes 2 and 3) or Kf (exo-) (lanes 5 and 6). Reactions in lanes 2 and 5 were incubated with dTTP,dGTP and ddATP. Reactions in lanes 3 and 6 were incubated with all four dNTPs. Incorporation of ddATP results in extension to a 36mer and incubation with all four dNTPs results in a 45mer product. (**C**) Strand displacement activity of EhDNApolA. Strand displacement was determined using a six-nucleotide substrate gap depicted below Figure3D. The reactions contained 25 units of φ 29 DNA polymerase (lane 2), 4 units of Taq DNA polymerase (lane 3), 5 units of T7 DNA polymerase (lane 4), and 45 fmol of EhDNApolA (lane 5). The 18mer primer can be freely extended to a 24mer. Further extension is only possible if the DNA polymerase displaced the annealed 21mer.

We tested the ability of EhDNApolA to shift a fixed amount of primer-template (3 nM) by increasing the EhDNApolA concentration from equimolar amounts to 60 fold excess (Figure 3A, lanes 2 to 7). The appearance of a major retarded band that increased in intensity according to the amount of added recombinant protein indicates that EhDNApolA is able to recognize a primer-template substrate. A few minor bands were also detected, however they had low abundance in comparison to the more abundant complex. It is possible that these bands resulted from some alternate binding mode of EhDNApolA to the primer-template, for instance a binding that resembled an editing complex [32], [33].

In order to test if recombinant EhDNApolA displays polymerization activity, we measured its ability to incorporate deoxynucleotides to an annealed primer-template. The presence of elongation products indicates that the recombinant EhDNApolA is a functional DNA polymerase (Figure 3B, lanes 2 and 3). The experimental setup placed the first template thymine at position 36 (Figure 3B). Thus, if dGTP, dCTP and ddATP were added as the only nucleotides in the reaction mixture, it is expected that elongation would stop at position 36. EhDNApolA readily incorporates ddATP and it is halted at position 36 (Figure 3B, lane 2). This is in contrast to Klenow fragment that did not efficiently incorporate ddATP, and replicates beyond the first thymine template (Figure 3B lane 5). Mutagenesis studies demonstrated that residue F762 of Klenow fragment is responsible for ddNTPs selectivity [34]. DNA polymerases with a tyrosine in the corresponding position incorporate ddNTP efficiently because the hydroxyl group of the tyrosine compensates for the missing 3′ OH of the ddNTPs [34]. The corresponding residue of Klenow fragment's F762 in EhDNApolA is a tyrosine (residue Y485). Thus, as it is observed, EhDNApolA efficiently incorporates ddNTPs during primer extension (Figure 3B lane 2). Several bands of lower molecular weight were observed during primer extension reactions. These bands may indicate that, like Klenow Fragment, EhDNApolA is a poorly processive DNA polymerase (Figure 3B, lanes 2–3 and 5–6).

Some family A DNA polymerases, like DNA polymerase γ and DNA polymerase ν are capable of strand displacement. We tested the strand displacement capabilities of EhDNApolA in comparison to other DNA polymerases. The strand displacement activity of EhDNApolA was measured in a primer-template substrate containing a gap of six nucleotides and this activity corresponds to the appearance of primer elongation products longer than 24nt (Figure 3C). φ29 DNA polymerase has strong strand displacement capabilities and is a highly processive polymerase. According to these characteristics, φ29 DNA polymerase is not halted at position 24 (Figure 3C, lane 2). Taq DNA polymerase and T7 DNA polymerase are DNA polymerases with moderate strand displacement, as some polymerase's population are blocked at positions 24 and 23 (Figure 3D, lanes 3 and 4). We found that EhDNApolA was able to perform strand displacement at similar levels that Taq DNA polymerase (Figure 3C, lanes 3 and 5). However, in contrast to Taq and T7 DNA polymerases, EhDNApolA has weak primer-template affinity during strand displacement, as evidenced by the apparition of bands from 25 to 30 nt (Figure 3C lane 5).We tested the ability of the purified EhDNApolA to degrade a labeled primer-template. No detectable 3′-5′ exonuclease activity was observed even after 8 minutes of incubation with EhDNApolA (data not shown). This is in agreement of our *in silico* prediction which indicates that EhDNApolA does not contain the motifs needed for 3′-5′ exonuclease activity [35].

### Kinetics parameters for EhDNApolA nucleotide incorporation

An important step to measure kinetic parameters is to determine the optimal reaction conditions. Thus, we determined the optimal salt concentration, pH, MgCl_2_ concentration, and temperature for EhDNApolA activity. EhDNApolA is strongly inhibited by NaCl. The optimal NaCl concentration for EhDNApolA activity is from 0 mM to 50 mM NaCl (Figure S1A, lanes 2 to 5). Increasing the NaCl concentration to 100mM only permitted the incorporation of a single nucleotide (Figure S1A, lane 6). EhDNApolA was not active at 200mM NaCl, a concentration that is similar to physiological concentrations. In this respect, EhDNApolA resembles Klenow fragment which has decreased activity at concentrations higher than 50 mM NaCl [36]. The optimal MgCl_2_ concentration was 2.5 mM (Figure S1B). This metal concentration was similar to the optimal concentration of *Thermus aquaticus* and Klenow Fragment DNA polymerases. The optimal pH for polymerization activity is 7.5. EhDNApolA has approximately 80% of activity between pH 7 and 8 (Figure S1C). As expected for an enzyme from a mesophilic organism, the optimal temperature for EhDNApolA activity was 37°C (Figure S1D). Using the optimal buffers, we determined the kinetic parameters for EhDNApolA activity using steady-state kinetics.

The K_m_ of the incoming nucleotide varied from 1.49 to 2.3 µM and the V_max_ varied between 2.9 to 3.3 nMol/min (Table S3). The kinetic constants of EhDNApolA were similar to several family A DNA polymerases including the DNA polymerase from *Bacillus stereothermophilu*s, Klenow Fragment and human DNA polymerase ν [20], [37], [38].

### EhDNApolA incorporates dNTPs with high selectivity

**Figure 4:**
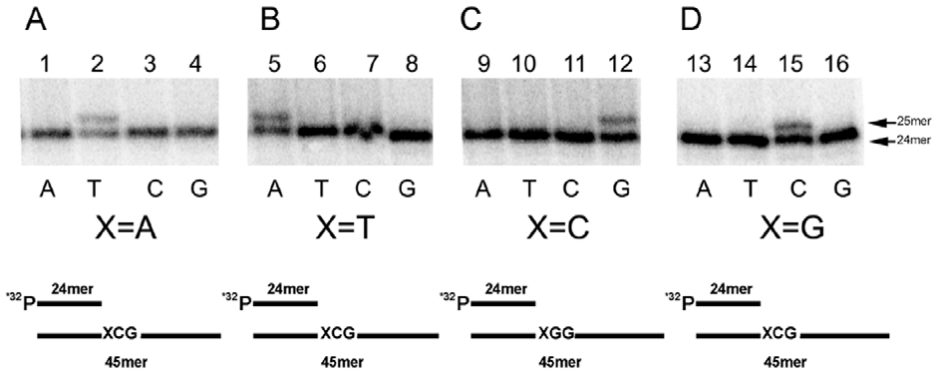
Nucleotide insertion fidelity of EhDNApolA. 16% denaturing polyacriylamide gel showing a primer-template extension by EhDNApolA in the presence of 100 µM of the indicated nucleotide. The first templated base is denoted with an X as depicted in figures A, B, C, and D. The identity of each dNTP in each reaction is indicated. The 24mer substrates and the 25mer products are indicated by an arrow. (**A**) Nucleotide fidelity for templated adenine (lanes 1 to 4). (**B**) Nucleotide fidelity for templated thymine (lanes 5 to 8). (**C**) Nucleotide fidelity for templated cytidine (lanes 9 to 12). (**D**) Nucleotide fidelity for templated guanine (lanes 13 to 16).

Family A DNA polymerases are highly variable in their DNA replication accuracy. Polymerases from bacteriophages, bacteria, and mitochondria are high fidelity polymerases. In contrast, human DNA polymerases θ and ν are low fidelity polymerases. For instance, human DNA polymerase ν misincorporates thymine across from a guanine template with a frequency of 0.45 [20]. To test the fidelity of EhDNApolA, we used a set of primer-templates in which the first template base is different from the following templated base (Figure 4). EhDNApolA selectively incorporated the incoming nucleotide according to the Watson-Crick rules at all four template bases (Figures 4A, 4B, 4C, and 4D). EhDNApol does not extensively misincorporate at canonical templates. This is in contrast to DNA polymerases of subfamilies ν and θ that are low fidelity polymerases. Although an extensive kinetic analysis is needed to quantify the fidelity of EhDNApol, it is evident that EhDNApol follows the Watson-Crick rules during nucleotide incorporation at canonical templates.

### Translesion DNA synthesis by EhDNApolA

**Figure 5:**
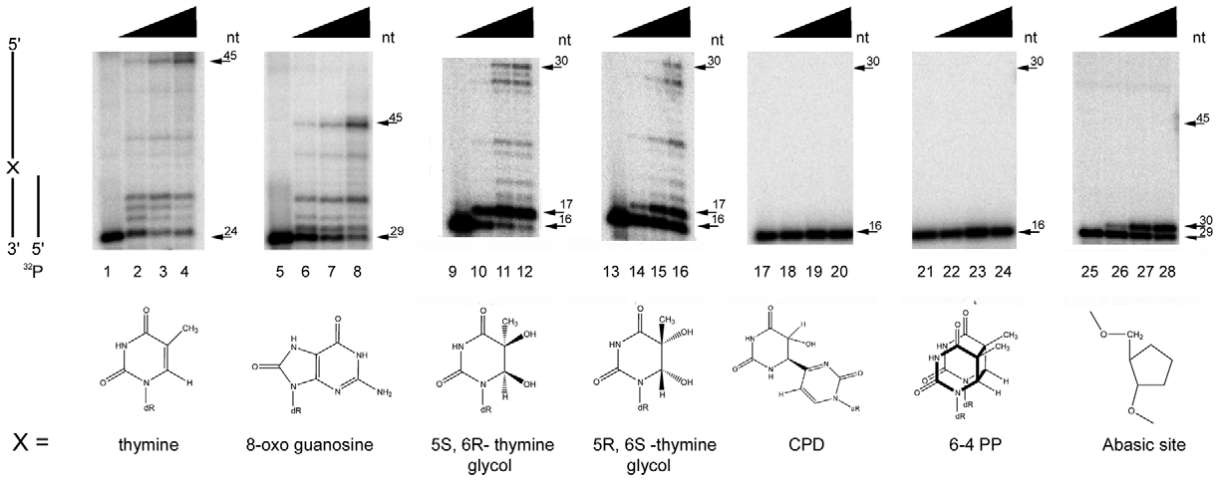
Translesion DNA synthesis by EhDNApolA. 16% denaturing polyacrylamide gel electrophoresis showing translesion bypass of EhDNApolA. The first templated base is denoted with an X. The structure of each templated lesion and a templated thymine are depicted for comparison. The reactions were incubated with increased amounts of EhDNApolA (0, 60, 120, and 240 fmol) and 20pM of each substrate. Thymine (lanes 1–4); 8-oxo guanosine (lanes 5–8,); 5 S-6R thymine glycol (lanes 9–12); 5R-6S thymine glycol (lanes 13–16,); cis-syn cyclobutane pyrimidine dimer (lanes 17–20); 6-4 photo product (lanes 21–24); abasic site (lanes 25–29). The bottom arrow indicates the substrate length and the top arrow indicates the expected full-length products.

DNA lesions can be classified as non-blocking and strong blocking. DNA lesions like 8-oxo guanosine are readily bypassed by the majority of family A DNA polymerases. On the other hand, DNA lesions like thymine glycol, abasic site, and thymine dimers are strong blocks to replication. Seeming exceptions are the cases of DNA polymerase ν that efficiently bypasses 5S-thymine glycol and DNA polymerase θ that bypasses abasic sites [19], [23]. To measure translesion DNA synthesis by EhDNApolA, we tested increasing amounts of the polymerase in a control template thymine and in several DNA lesions. To permit the relative extension comparison, less than 50% of the control thymine template was extended at the lower polymerase concentration. EhDNApolA extended a thymine template to the final 45mer product with an efficiency of 42% at the higher polymerase concentration (Figure 5, lane 4). EhDNApolA efficiently bypasses 8-oxo guanosine, as 26% of the labeled primer was extended to the final 45mer product at the higher polymerase concentration (Figure 5, lanes 5 to 8). EhDNApolA bypasses 5S, 6R thymine glycol with an efficiency of 6%, this efficiency is low in comparison to the thymine template, but is significantly larger than other DNA polymerases, like RB69 that which is completely blocked by this lesion [20], [39]. The stalled 17mer product constitutes 80% of the total labeled DNA in the reaction (Figure 5, lanes 9 to 12). Similar results have been observed for an exonuclease deficient Klenow fragment [20], [40]. EhDNApolA bypasses the 5R, 6S thymine glycol with an efficiency of 4% (Figure 5, lanes 13 to 16). As in the case of the 5S, 6R thymine glycol lesion, this efficiency was low in comparison to the control thymine but is more efficient than DNA polymerase ν [20] or any other family A DNA polymerase characterized to date. The stalled 17mer product represents 33% of the product (Figure 5, lane 16). EhDNApolA is unable to bypass the CPD and the 6-4 photoproduct (Figure 5, lanes 17 to 24). EhDNApolA incorporates only one nucleotide opposite an abasic site (Figure 5, lanes 25 to 28) and some bypass occurs at higher polymerase concentrations (data not shown).

### EhDNApolA bypasses 8-oxoguanosine with low fidelity and follows the “A rule” during dNTP incorporation across from an abasic site

**Figure 6:**
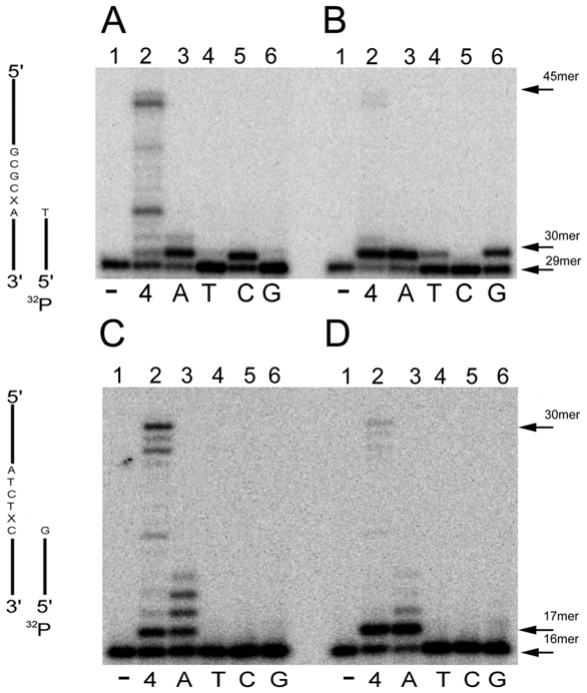
Fidelity of translesion DNA synthesis by EhDNApolA. 16% denaturing polyacrylamide gel electrophoresis showing translesion bypass fidelity of EhDNApolA. 0.2 pmol of EhDNApolA were incubated with a set of substrates containing several DNA lesions. The reactions were carried out with four dNTPs or single dNTP addition. (**A**) 8-oxo guanosine (lanes 1 to 6). (**B**) abasic site (lanes 1 to 6). (**C**) 5 S-6R thymine glycol (lanes 1 to 6). (**D**) 5R-6S thymine glycol (lanes 1 to 6). The identity of each nucleotide is indicated in the figure. The length of the substrates, single nucleotide extensions and full-length products are indicated by arrows.

EhDNApolA was able to bypass 8-oxoguanosine and to incorporate across from an abasic site (Figure 5, lanes 6 to 8 and 26 to 28). In order to test the fidelity of lesion bypass, we tested the incorporation of each deoxyribonucleotide across from each lesion. 8-oxoguanosine is a dual code lesion that can template for dCTP and dATP. The *syn* conformation of 8-oxoguanosine mimics a thymine template that allows dATP incorporation[41]. EhDNApolA incorporated dATP across from 8-oxoguanosine more efficiently than dCTP (Figure 6A, lanes 2 and 5). The rationale for this incorporation resides in the nature of a specific residue at the fingers subdomain. A bulky residue like K635 in T7 DNApol dictates the incorporation of dCTP [41] whereas a glycine residue in *B. stearothermophilus* DNA polymerase may dictate the incorporation of dATP [38], [42]. EhDNApolA contains a serine in the position corresponding to residue K635 of T7 DNA polymerase, thus the preferential incorporation of dATP is predicted. Family A DNA polymerases preferentially insert dATP across from an abasic site, a phenomena known a as the “A-rule” [43]. EhDNApolA incorporates preferentially dATP (Figure 6B, lane 3) and dGTP (Figure 6B, lane 6) opposite abasic sites. EhDNApolA only incorporates a purine across from the lesion, but it does not extend from the lesion (Figure 6B). This characteristic is conserved with other family A DNA polymerases, like Klenow fragment [44] or DNA polymerase ν [20]. However, DNA polymerase θ is able to bypass abasic sites [23]

### EhDNApolA bypasses thymine glycol with high fidelity

In contrast to replicative DNA polymerases, like DNA polymerase RB69, that stall at thymine glycol lesion, EhDNApol is able to bypass this lesion. Family A DNA polymerases, like an exonuclease deficient Klenow fragment bypasses the 5S, 6R thymine glycol lesion, but are halted at the 5R, 6S-thymine glycol lesion [20]. Although EhDNApol readily incorporates across from a 5S, 6R thymine glycol lesion, it is severely hampered during its elongation. The 5R, 6S thymine glycol lesion is also bypassed by EhDNApol, although with different properties than the 5S, 6R thymine glycol lesion. The accumulation of the first incorporated nucleotide occurs less efficiently than in the 5S, 6R lesion. Structural studies suggest that thymine glycol prevents primer extension by obstructing the next 5′ templated base to stack against it [39].

EhDNApolA is able to accurately bypass thymine glycol (Figures 6C and 6D). EhDNApolA inserts dATP opposite 5S, 6R thymine glycol (Figure 6C, lane 3) and 5R, 6S thymine glycol (Figure 6D, lane 3). EhDNApolA did not incorporate any other nucleotide opposite 5S, 6R or 5R, 6S thymine glycol (Figure 6C, lanes 4 to 6 and Figure 6D, lanes 4 to 6). EhDNApolA incorporates dATP at the 5S, 6R thymine glycol lesion and in this context misincorporates dATP opposite template dCTP. A similar phenomenon occurs in human DNA polymerase κ [40] indicating that a subtle DNA distortion originated by the lesion may influence nucleotide incorporation fidelity by these DNA polymerases, as observed by the apparent low fidelity of EhDNApolA. This is in contrast to the high fidelity opposite template dCTP in a canonical template (Figure 4C).

### Nuclear localization of EhDNApolA

**Figure 7:**
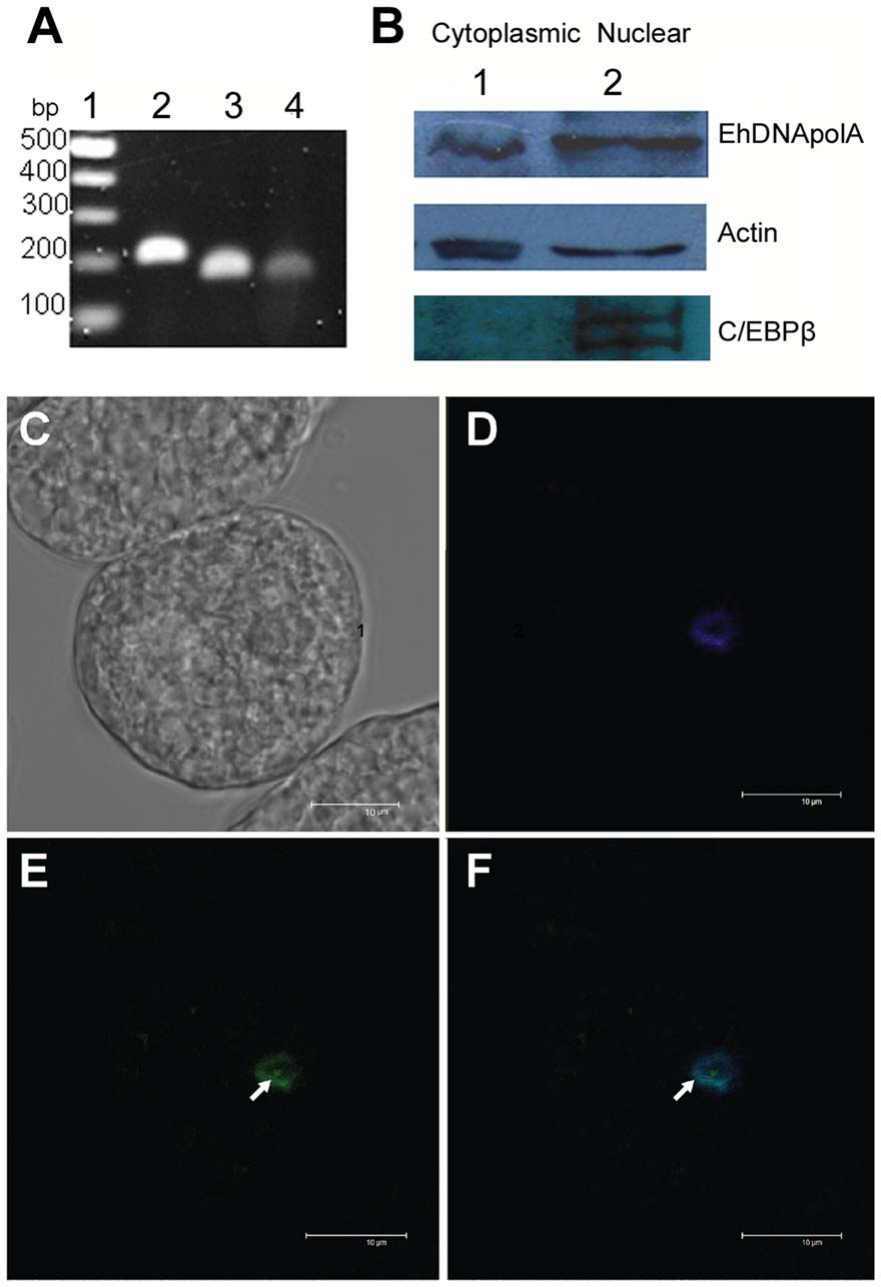
Cellular identification and localization of EhDNApolA. (**A**) RT-PCR using total RNA from *E. histolytica* trophozoites grown in basal culture conditions 1% Agarose gel stained with ethidium bromide showing the RT-PCR products of motifs A (lane 3) and C (lane 4) of the EhDNApolA gene. The RT-PCR product of the actin gene control is shown in lane 2. (**B**) Immunodetection of EhDNApolA. Western blot assays using, cytoplasmic extracts (lane 1), and nuclear extracts (lane 2) of *E. histolytica*'s trozophoites against mouse polyclonal anti- EhDNApolA antibodies (upper pannel). Control using mouse polyclonal anti-actin antibodies (middle panel) and anti- C/EBPβ antibodies (lower panel) (**C–F**) Cellular localization of EhDNApolA analyzed by confocal immunofluorescence microscopy. (**C**) Nomarsky image of a single *E. histolytica* cell stained with 4′,6-diamidino-2-phenylindole (DAPI) and incubated with anti-EhDNApolA antibodies treated with FITC-labeled secondary antibodies (**D**) fluorescence signal generated by DAPI (**E**) fluorescence signal generated by the binding of the FITC-conjugated antibody to anti-EhDNApol A antibody (**F**) Merge image of the fluorescence emitted by DAPI and FITC.

In order to verify that the gene of EhDNApolA is transcribed *in vivo*, we carried out a RT-PCR using specific oligonucleotides that amplified the two conserved motifs A and C of the EhDNApolA gene. The oligonucleotides were designed to amplify a region of 168 bp corresponding to motif A and a region of 156 bp corresponding to motif C of the EhDNApolA gene (Figure 7A, lanes 3 and 4 respectively). The RT-PCR control product of the actin gene control corresponds to 192 bp (Figure 7A, lane 2) and the no RT reaction showed no appearance of a new band (data not shown). The RT-PCR reaction produced the expected products, thus confirming that the EhDNApolA gene is transcribed under basal conditions in *E. histolytica*. In order to quantify the abundance of the EhDNApolA transcript, we compared the relative transcript under basal conditions in comparison to actin. The average intensity of the EhDNApolA transcript is approximately 70% of the intensity of the actin transcript (Figure S2). Thus, the EhDNApol A gene is expressed at similar levels than the actin gene in basal cell culture conditions. To determine the localization of EhDNApolA in *E. histolytica,* we carried out Western blot analyses of fractionated cytoplasm and nuclear extracts using the anti-peptide EhDNApol A antibody and anti-actin and anti-C/EBPβ antibodies as controls. The appearance of a single protein band of 75kDa in the nuclear and cytoplasmic fractions using the anti-peptide EhDNApol A antibody indicates that a population of EhDNApolA is translocated from the cytoplasm into the nucleus (Figure 7B, lanes 1 and 2). The same patter is observed with the anti-actin antibody, as actin is a protein with cytoplasmic and nuclear localization [30]. Because nuclear fractions are often contaminated with cytosolic fractions, we used the identification of C/EBPβ, as a control of the nuclear extract purification protocol. The antibody against this protein identifies a double band of approximately 65 kDa in Western blot assays; however this recognition occurs predominantly in nuclear extracts and not in the cytoplasmic fraction [45]. The data indicates that a population of EhDNApolA is imported from the cytoplasm into the nucleus. Confocal microscopy of *E.histolytica* trophozoites stained with antibodies against the peptide of EhDNApolA corroborates that EhDNApolA is translocated into the nucleus (Figure 7). DAPI staining indicates the localization of nuclear double-stranded DNA in the parasite (Figure 7D) and immunofluorescence analysis using anti-EhDNApolA antibodies shown a possible nuclear localization (Figure 7E). Merged field indicated that EhDNApol A colocalizes with DAPI staining of the nuclear DNA of *E. histolytica* (Figure 7 F). An analysis of the EhDNApolA amino acid sequence using the pSORT program (http://psort.ims.u-tokyo.ac.jp/) predicted the presence of several nuclear localization signals. DNA polymerization in *E. histolytica* is inhibited by aphidicolin, which is an inhibitor of family B DNA polymerases and is weakly inhibited by ddNTPs [46], [47]. As EhDNApolA readily incorporates ddNTPs (Figure 3B) and family A DNA polymerase are not inhibited by aphidicolin, EhDNApolA should not play a preponderant role in DNA replication of *E. histolytica*'s genome.

## Discussion

In this work we report the cloning and biochemical characterization of a family A DNA polymerase present in *E. histolytica.* Although *E. histolytica* contains a mitocondrial remnant organelle dubbed mitosome, this organelle does not contain DNA. Furthermore, the genome of *E. histolytica* does not contains a phage-type RNA polymerase and DNA helicase involved in transcription and replication of mitochondrial DNA [9]. EhDNApolA may have evolved from the ancestral mitochondrial DNA polymerase γ or was acquired by horizontal gene transfer from a bacterial family A DNA polymerase. The fact that EhDNApolA is biochemically related to DNA polymerase ν may be a case of convergent evolution as DNA polymerases of subfamily N are only present in vertebrates [19], [48]. Thymine glycol is a DNA lesion formed by chemical oxidation and ionizing radiation [49]. *E. histolytica* is subject to reactive oxygen species produced at the colonic tissue and by phagocyte release [4], [50]. In eukaryotic organisms, thymine glycol can be bypassed by DNA polymerases κ and η [40], [51]. However, *E. histolytica* lacks those DNA polymerases. *E. histolytica* contains genes for Base Excision Repair including functional 8-oxo guanosine and thymine glycol glycosylases (Garcia et al, manuscript in preparation). Although, the *in vivo* function of EhDNApolA is unknown, its abilities to bypass thymine glycol and nuclear localization suggest a possible role of this enzyme in translesion DNA synthesis. This role is reminiscent of family A DNA polymerases of *Arabidopsis thaliana* postulated to be involved in DNA repair at the chloroplast [52] and eukaryotic family A DNA polymerases ν and θ [19], [24].

## Supporting Information

Figure S1 Optimal activity conditions of EhDNApolA. To determine the optimal conditions for DNA polymerization, 60 fmol of EhDNApolA were incubated with 200 fmol of nicked substrate for 10 min under varying experimental conditions. DNA polymerase activity was measured by the extension of a 24 mer primer to a 45mer product (A) Effect of salt on DNA polymerization activity. Lane1 corresponds to negative control with no polymerase, lane 2 no added salt salt, lanes 3 to 8 NaCl concentrations from 12.5 mM to 400 mM (B) MgCl2 dependence of EhDNApolA activity. The values were normalized to 100% as the higher polymerization value and the subsequent values were calculated as a relative percentage. The solid bars represent the relative percentage of polymerization. (C) pH influence on DNA polymerization activity (D) Temperature dependence of EhDNApolA activity.
(0.14 MB TIF)

Figure S2 mRNA expression profiles of the EhDNApolA gene. RT-PCR analysis of EhDNApolA (Upper panel) in comparison to actin (lower panel). Amplification products using isolated RNA treated with Reverse Transcriptase (RT +) (lanes 1 to 3) or with-out Reverse Transcriptase (RT -) (data not shown) were run on a 1% agarose gel and stained with ethidium bromide. The densitometric analysis of the RT-PCR product corresponding to the actin gene was designated as 100%. The EhDNApolA gene is expressed 71% of the actin control. Standard deviations were calculated based on three independent experiments.
(0.49 MB TIF)

Table S1 Putative DNA polymerases present in the genome of *E. histolytica*. This table presents a comparison between the putative DNA polymerases present in the genome of *E. histolytica* and homologous DNA polymerases.
(0.05 MB DOC)

Table S2 GenBank accession numbers of the DNA polymerases used in the phylogenetic tree. This table contains the GenBank accession numbers of the DNA polymerases used to build the phylogenetic tree.
(0.07 MB DOC)

Table S3 Kinetic parameters for nucleotide incorporation by EhDNApolA. This table contains the kinetics parameters (Km and Vmax) for nucleoide incorporation of EhDNApolA.
(0.03 MB DOC)
