# Supplementary figures and images for: A Nuclear Family A DNA Polymerase from Entamoeba histolytica Bypasses Thymine Glycol
Source: PLoS Negl Trop Dis. 2010 Aug 10;4(8):e786. doi: 10.1371/journal.pntd.0000786 (PMC2919377; doi:10.1371/journal.pntd.0000786)

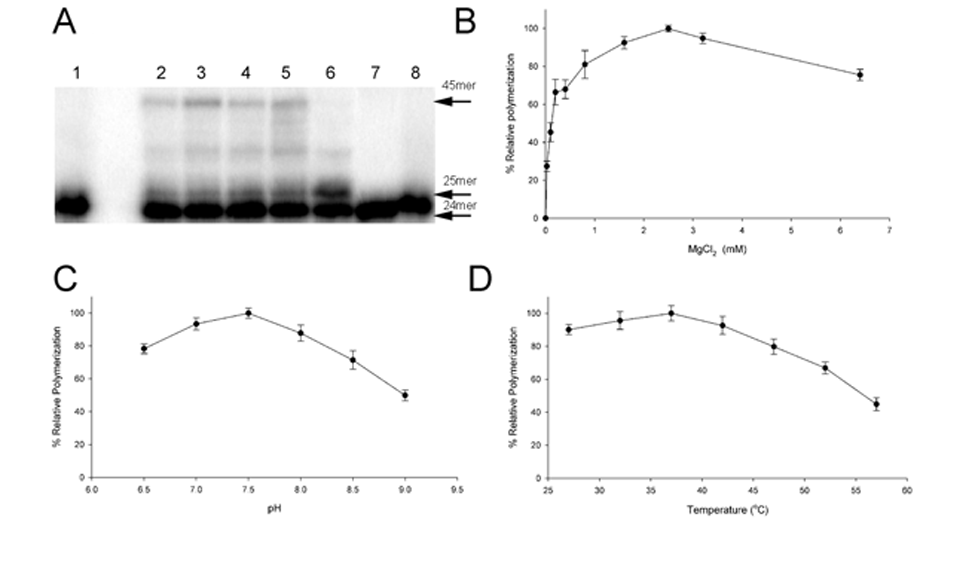

Supplement: Figure S1 — Optimal activity conditions of EhDNApolA. To determine the optimal conditions for DNA polymerization, 60 fmol of EhDNApolA were incubated with 200 fmol of nicked substrate for 10 min under varying experimental conditions. DNA polymerase activity was measured by the extension of a 24 mer primer to a 45mer product (A) Effect of salt on DNA polymerization activity. Lane1 corresponds to negative control with no polymerase, lane 2 no added salt salt, lanes 3 to 8 NaCl concentrations from 12.5 mM to 400 mM (B) MgCl2 dependence of EhDNApolA activity. The values were normalized to 100% as the higher polymerization value and the subsequent values were calculated as a relative percentage. The solid bars represent the relative percentage of polymerization. (C) pH influence on DNA polymerization activity (D) Temperature dependence of EhDNApolA activity. (0.14 MB TIF) [file pntd.0000786.s001.tif]

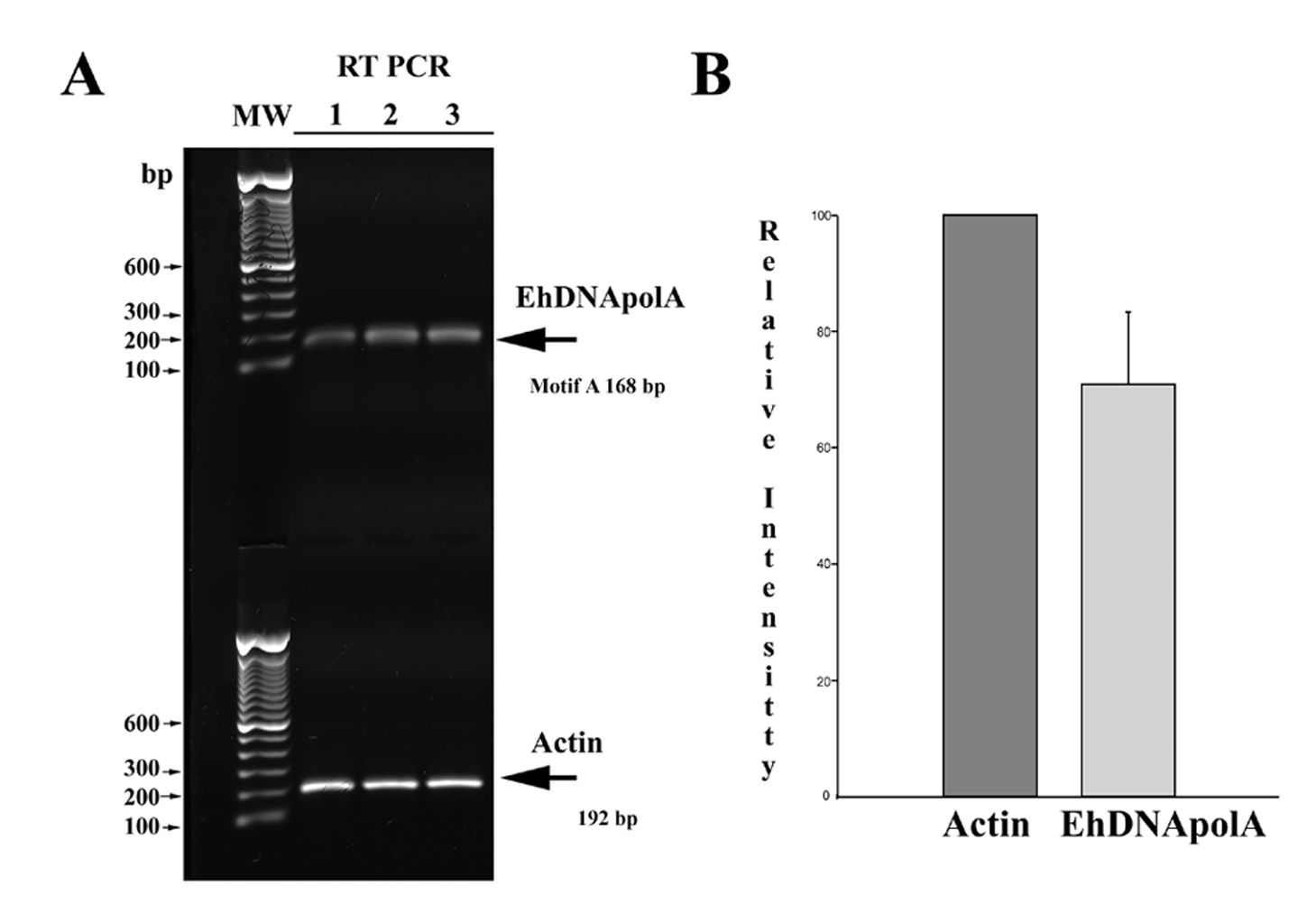

Supplement: Figure S2 — mRNA expression profiles of the EhDNApolA gene. RT-PCR analysis of EhDNApolA (Upper panel) in comparison to actin (lower panel). Amplification products using isolated RNA treated with Reverse Transcriptase (RT +) (lanes 1 to 3) or with-out Reverse Transcriptase (RT -) (data not shown) were run on a 1% agarose gel and stained with ethidium bromide. The densitometric analysis of the RT-PCR product corresponding to the actin gene was designated as 100%. The EhDNApolA gene is expressed 71% of the actin control. Standard deviations were calculated based on three independent experiments. (0.49 MB TIF) [file pntd.0000786.s002.tif]
