# Supplementary material for: A Nuclear Family A DNA Polymerase from Entamoeba histolytica Bypasses Thymine Glycol
Source: PLoS Negl Trop Dis. 2010 Aug 10;4(8):e786. doi: 10.1371/journal.pntd.0000786 (PMC2919377; doi:10.1371/journal.pntd.0000786)
Supplement: Table S1 — Putative DNA polymerases present in the genome of E. histolytica. This table presents a comparison between the putative DNA polymerases present in the genome of E. histolytica and homologous DNA polymerases. (0.05 MB DOC) [file pntd.0000786.s003.doc]

Supporting Table S1 Putative DNA polymerases present in the genome o*f E. histolytica*

| *Gene* | *aa length* | *Exonuclease domain* | *GenBank ID* | *Pathema Locus* | *Polymerase*  *family* | *Putative Function* | *Related DNA Polymerase* | *GenBank ID* |
| --- | --- | --- | --- | --- | --- | --- | --- | --- |
| EhDNAPOLA1 | 1133 | + | XP_657373.1 | EHI_151520 | B | DNA replication | HsPOLA1  (catalytic subunit) | NP_058633 |
| EhDNAPOLD1 | 1078 | + | XP_654477.1 | EHI_006690 | B | DNA replication | HsPOLD1 (catalytic subunit) | NP_002682 |
| EhDNAPOLE1 | ?? | ?? | ?? | ?? | ?? | DNA replication | HsPOLE1 (catalytic subunit) | NP_006222.2 |
| EhREV1 | 709 | - | XP_654241.1 | EHI_053480 | Y | translesion DNA synthesis | HsREV1 | NP_057400.1 |
| EhREV3 | 1386 | - | XP_656768 | EHI_068010 | B | translesion DNA synthesis | HsREV3 | NP_002903 |
| EhDNApolA | 657 | - | XP_653960 | EHI_073640 | A | translesion DNA synthesis | HsPOLN | AAN52116 |
| EhDNAPOLB1 | 699 | + | XP_001914313 | EHI_037680 | B2 | transposable element | DNA polφ29 | YP_002004529 |
| EhDNAPOLB2 | 813 | + | XP_001914292 | EHI_018010 | B2 | transposable element | DNA polφ29 | YP_002004529 |
| EhDNAPOLB3 | 1080 | + | XP_649845 | EHI _132860 | B2 | transposable element | DNA polφ29 | YP_002004529 |
| EhDNAPOLB4 | 1279 | + | XP_001913700 | EHI_164190 | B2 | transposable element | DNA polφ29 | YP_002004529 |
| EhDNAPOLB5 | 1231 | + | XP_648196 | EHI_196700 | B2 | transposable element | DNA polφ29 | YP_002004529 |
|  |  |  |  |  |  |  |  |  |
