# Supplementary material for: A Nuclear Family A DNA Polymerase from Entamoeba histolytica Bypasses Thymine Glycol
Source: PLoS Negl Trop Dis. 2010 Aug 10;4(8):e786. doi: 10.1371/journal.pntd.0000786 (PMC2919377; doi:10.1371/journal.pntd.0000786)
Supplement: Table S2 — GenBank accession numbers of the DNA polymerases used in the phylogenetic tree. This table contains the GenBank accession numbers of the DNA polymerases used to build the phylogenetic tree. (0.07 MB DOC) [file pntd.0000786.s004.doc]

**Supporting TableS2.** GenBank accession numbers of the polymerases used to build the phylogenetic tree

| Abbreviation | Organism | Accession number |
| --- | --- | --- |
| *E. histolytica* | *Entamoeba histolytica* | XP_653960 |
| *E. dispar* | *Entamoeba dispar* | XP_001739984 |
| *E. invadens* | *Entamoeba invadens* | EIN_094210 |
| *B. avium* | *Bordetella avium* | YP_786902 |
| *F. tularensis* | *Francisella tularensis* | YP_001891039 |
| *R. erythropolis* | *Rhodococcus erythropolis* | AAG43148 |
| *N. gonorrhoeae* | *Neisseria gonorrhoeae* | YP_209116 |
| *C. botulinum* | *Clostridium botulinum* | ZP_02622469 |
| *B. valaisiana* | *Borrelia valaisiana* | ZP_03672778 |
| *P. aeruginosa* | *Pseudomonas aeruginosa* | NP_254180 |
| *T. maritima* | *Thermotoga maritima* | NP_229419 |
| *N. crassa* | pol G *Neurospora crassa* | AAD21034 |
| *S. pompe* | pol G *Schizosaccharomyces pombe* | CAA88012 |
| *S. cerevisiae* | pol G *Saccharomyces cerevisiae* | CAA99652 |
| *P. pastoris* | pol G *Pichia pastoris* | XP_002492933 |
| *H. sapiens* | pol G *Homo sapiens* | NP_002684 |
| *G. gallus* | pol G *Gallus gallus* | Q92076 |
| *D. melanogaster* | pol G *Drosophila melanogaster* | AAL89964 |
| *Bacteriophage T7* | *Enterobacteria phage T7* | NP_041982 |
| *Bacteriophage T3* | *Enterobacteria phage T3* | NP_523320 |
| *Bacteriophage phiA1122* | *Yersinia pestis phage phiA1122* | NP_848283 |
| *H. Sapiens* | *pol N Homo sapiens* | NP_861524.2 |
| *D. radiodurans* | *Deinococcus radiodurans* | NP_295430 |
| *T. aquaticus* | *Thermus aquaticus* | P19821 |
| *H. influenzae* | *Haemophilus influenzae* | NP_439016 |
| *M. tuberculosis* | *Mycobacterium tuberculosis* | NP_216145 |
| *E. coli* | *Escherichia coli* | NP_290488 |
| *T. thermophilus* | *Thermus thermophilus* | P30313 |
| *B. subtilis* | *Bacillus subtilis* | NP_390787 |
| *S. aureus* | *Staphylococcus aureus* | NP_372214 |
| *M. Musculus* | pol N *Mus musculus* | NP_862905 |
| *G. gallus* | pol N *Gallus gallus* | XP_420834 |
| *D. rerio* | pol N *Danio rerio* | NP_001093496 |
| *C. familiaris* | pol N *Canis familiaris* | XP_545922 |
| *R. norvegicus* | pol N *Rattus norvegicus* | XP_001065133 |
| *B. taurus* | pol N *Bos taurus* | XP_601658 |
| *E. Caballus* | pol N Equus caballus | XP_001489138 |
