# Supplementary material for: A Nuclear Family A DNA Polymerase from Entamoeba histolytica Bypasses Thymine Glycol
Source: PLoS Negl Trop Dis. 2010 Aug 10;4(8):e786. doi: 10.1371/journal.pntd.0000786 (PMC2919377; doi:10.1371/journal.pntd.0000786)
Supplement: Table S3 — Kinetic parameters for nucleotide incorporation by EhDNApolA. This table contains the kinetics parameters (Km and Vmax) for nucleoide incorporation of EhDNApolA. (0.03 MB DOC) [file pntd.0000786.s005.doc]

**Supporting Table S3** Kinetics parameters for nucleotide incorporation by EhDNApolA

| DNA substrate | dNTP | K_m_ ( µM) | V_max_ (nMol/min) |
| --- | --- | --- | --- |
|  | dATP | 2.3 ± 0.2 | 3.3 ± 0.4 |
| 5' ATG  TACX | dCTP | 2.1 ± 0.3 | 3.2 ± 0.4 |
|  | dGTP | 1.56 ± 0.2 | 3.5 ± 0.3 |
|  | dTTP | 1.49 ± 0.3 | 2.9 ± 0.2 |
